# Памяти Эльвиры Петровны Касаткиной (27 марта 1930 г. – 18 апреля 2022 г.)

**DOI:** 10.14341/probl13116

**Published:** 2022-04-30

**Keywords:** Эльвира Петровна Касаткина, эндокринология

## Abstract

18 апреля 2022 года на 93 году жизни скончалась заслуженный врач Российской Федерации, почетный заведующий кафедрой детской эндокринологии РМАНПО, доктор медицинских наук, профессор Эльвира Петровна Касаткина.

## Abstract (en)

On April 18, 2022, Professor Elvira Petrovna Kasatkina, Honored Doctor of the Russian Federation, Honorary Head of the Department of Pediatric Endocrinology of the RMANPO, Doctor of Medical Sciences, died at the age of 93.

18 апреля 2022 года на 93-м году жизни скончалась заслуженный врач Российской Федерации, почетный заведующий кафедрой детской эндокринологии РМАНПО, доктор медицинских наук, профессор Эльвира Петровна Касаткина.

Родилась Эльвира Петровна в городе Астрахань, после окончания в 1955 году медицинского института работала врачом-педиатром, а затем продолжила обучение в клинической ординатуре в НИИ экспериментальной эндокринологии и химии гормонов АМН СССР.

Эльвира Петровна прошла путь от врача педиатра до заведующей кафедрой детской эндокринологии в Российской медицинской академии непрерывного профессионального образования, с которой связана вся ее профессиональная деятельность, начиная с 1962 года.

В 1966 году Эльвира Петровна успешно защитила кандидатскую диссертацию на тему «Врожденная дисфункция коры надпочечников у детей: клинико-патогенетическая характеристика, диагностика, лечение», а в 1977 году — докторскую диссертацию на тему «Дифференциальная диагностика и терапия гермафродитизма».

Талантливый педагог, ученый и организатор, Касаткина Э.П. отдавала все свои знания, способности и энергию важному делу – подготовке детских эндокринологов.

В течение долгих лет Эльвира Петровна являлась главным внештатным детским специалистом эндокринологом Минздрава России, именно по ее инициативе в 1997 году в номенклатуру медицинских специальностей была включена отдельная специальность «детская эндокринология».

Касаткина Э.П. — автор свыше 400 научных работ, нормативных и учебно-методических пособий, 5 патентов на изобретения, 7 монографий, в том числе первой в стране монографии «Сахарный диабет у детей и подростков», которая многократно переиздавалась и до настоящего времени является настольной книгой как врачей, так и пациентов. Эльвирой Петровной было подготовлено 29 кандидатов наук и 10 докторов наук.

Во всех уголках нашей страны и за рубежом работают ее ученики, многие из них — главные внештатные детские специалисты эндокринологи регионов.

Эльвира Петровна вела большую общественную работу, являлась членом редколлегии журналов «Сахарный диабет», «Проблемы эндокринологии», «Ожирение и метаболизм », членом Диссертационного совета Эндокринологического научного центра и ФГБОУ ДПО РМАНПО по специальности «Эндокринология».

Заслуги Касаткиной Э.П. отмечены почетными дипломами ВДНХ, благодарностью Министерства здравоохранения, нагрудным знаком «Отличник здравоохранения», премией города Москвы 2007 года в области медицины, почетными грамотами Минздрава России.

Детская эндокринология понесла невосполнимую утрату, ушел из жизни великолепный врач, талантливый педагог, мудрый наставник, Учитель с большой буквы. Память о Касаткиной Эльвире Петровне навсегда останется в наших сердцах.

Редакционная коллегия журнала «Проблемы эндокринологии » присоединяется к соболезнованиям.

